# Accumulation and Phosphorylation of RecQ-Mediated Genome Instability Protein 1 (RMI1) at Serine 284 and Serine 292 during Mitosis

**DOI:** 10.3390/ijms161125965

**Published:** 2015-11-04

**Authors:** Chang Xu, Yan Wang, Lu Wang, Qin Wang, Li-Qing Du, Saijun Fan, Qiang Liu, Lei Li

**Affiliations:** 1 Tianjin Key Laboratory of Radiation Medicine and Molecular Nuclear Medicine, Institute of Radiation Medicine, Chinese Academy of Medical Sciences and Peking Union Medical College, Tianjin 300192, China; xuchang@irm-cams.ac.cn (C.X.); wangyan@irm-cams.ac.cn (Y.W.); lilithwww@sina.com (L.W.); wangqin@irm-cams.ac.cn (Q.W.); duliqing@irm-cams.ac.cn (L.-Q.D.); fansaijun@irm-cams.ac.cn (S.F.); 2 Department of Experimental Radiation Oncology, the University of Texas MD Anderson Cancer Center, Houston, TX 77030, USA

**Keywords:** RMI1 (RecQ-mediated genome instability protein 1), BTR (BLM-Topo IIIα-RMI) complex, mitosis, phosphorylation

## Abstract

Chromosome instability usually leads to tumorigenesis. Bloom syndrome (BS) is a genetic disease associated with chromosome instability. The BS gene product, BLM, has been reported to function in the spindle assembly checkpoint (SAC) to prevent chromosome instability. BTR complex, composed of BLM, topoisomerase IIIα (Topo IIIα), RMI1 (RecQ-mediated genome instability protein 1, BLAP75) and RMI2 (RecQ-mediated genome instability protein 2, BLAP18), is crucial for maintaining genome stability. Recent work has demonstrated that RMI2 also plays critical role in SAC. However, little is know about RMI1 regulation during the cell cycle. Here we present that RMI1 protein level does not change through G1, S and G2 phases, but significantly increases in M phase. Moreover, phosphorylation of RMI1 occurs in mitosis. Upon microtubule-disturbing agent, RMI1 is phosphorylated primarily at the sites of Serine 284 and Serine 292, which does not interfere with the formation of BTR complex. Additionally, this phosphorylation is partially reversed by roscovitine treatment, implying cycling-dependent kinase 1 (CDK1) might be one of the upstream kinases.

## 1. Introduction

Bloom syndrome (BS) is a severe chromosome instability disease characterized by predisposition to a wide range of cancers and around 10-fold elevation of sister chromatid exchanges (SCEs) frequency in BS cells [1,2]. BS is caused by one mutated gene, BLM, which is one of members of the RecQ DNA helicase family [3]. BLM has been extensively demonstrated to play multiple DNA transaction roles in DNA replication, repair, and recombination [4,5].

BLM has been reported to physically and/or functionally interact with a number of proteins, such as DNA damage checkpoint kinases ATM (Ataxia telangiectasia mutated) and ATR (ATM and Rad3-related) [6]; homologous recombination protein RAD51 recombinase [7]; mismatch repair protein MLH1 (MutL Homolog 1) and MSH6 (MutS Homolog 6) [8,9]; DNA replication checkpoint protein TopBP1 (topoisomerase-IIβ-binding protein 1) [10]; telomere-binding protein TRF2 (telomere repeat binding factor 2) [11]. Although the list of BLM protein partners is becoming progressively lengthier, it was found that BLM only has three consistent protein partners, Topo IIIα (topoisomerase IIIα), RMI1 (RecQ-mediated genome instability protein 1, BLAP75), and RMI2 (RecQ-mediated genome instability protein 2, BLAP18), which form a BTR complex with BLM [12,13,14]. Both RMI1 and RMI2 contain OB-fold (oligonucleotide/oligosaccharide binding) domain. In BTR complex, RMI1 directly binds to Topo IIIα while RMI2 interacts directly with BLM [15]. The BTR complex has been shown to play a critical role in resolving double Holliday junctions during the process of homologous recombination [16,17,18]. All members of the BTR complex are essential for maintaining genomic stability [14,19].

Previous studies have established that BLM protein levels are regulated in a cell cycle-dependent manner. In G1 phase, the BLM protein level is relatively low. During S and G2/M phases, the amount of BLM protein increases significantly [20]. The fluctuating BLM protein level is consistent with BLM functions in DNA replication, homologous recombination, and preventing sister chromatid exchanges, since all of these events could only occur in S, G2, and M phases. At the post-translational modification level, it has been found that BLM and RMI2 are phosphorylated upon spindle assembly checkpoint (SAC) activation, which is dependent on the mitotic kinase monopolar spindle 1 (MPS1). Functionally, phosphorylation of BLM on Ser144 and phosphorylation of RMI2 on Ser112 are important for maintaining mitotic arrest and genomic stability [21,22]. RMI1 is an integral and functional component of the BTR complex [12]. However, little is known about whether RMI1 is regulated or modified in cell cycle. Here, we determined the protein levels of RMI1 through the cell cycle. In addition, our studies reveal that RMI1 is phosphorylated with similar kinetics to BLM during mitosis, with two major phosphorylation sites, Ser284 and Ser292.

## 2. Results

### 2.1. RMI1 (RecQ-Mediated Genome Instability Protein 1) Protein Level Remains Unchanged through the Cell Cycle G1 to G2 Phase

**Figure 1:**
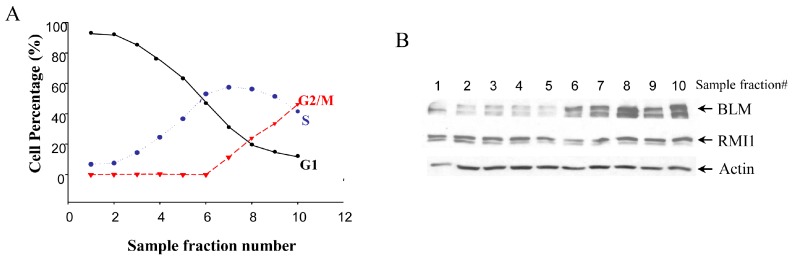
Levels of RMI1 protein (RecQ-mediated genome instability protein 1) in cycling HeLa cells. (**A**) HeLa cells were synchronized by centrifugal elutriation. The cell cycle distribution of each fraction was determined by flow cytometry; (**B**) Total proteins were extracted from each fraction cells and analyzed for protein levels of RMI1 and BLM by immunoblotting. Numbers above the panel denote individual elutriated fractions.

Given that BLM protein level is cell cycle-regulated, we examined whether there are any cell cycle-specific changes in RMI1 expression level by using synchronized cells at various cell cycle stages. Treating cells with different drugs is widely used in cell synchronization. However, this method may induce DNA damage, which might result in RMI1 protein alteration. To minimize the perturbation of cell physiological status by chemical-based synchronization methods, cell cycle stage-specific cells were obtained by centrifugal elutriation of cycling HeLa cells. This mechanical method allows cells to be fractionated on the basis of their sedimentation velocity [23]. The smaller G1 cells are elutriated first, followed by S phase cells and lastly the biggest G2/M cells. As such, it is possible to obtain a large amount of nearly synchronous cells without chemically interfering with the cell cycle. The degree of synchrony of each fraction was assessed by flow cytometric analysis (Figure 1A). The lysate of each fraction was analyzed by Western blotting using both anti-RMI1 and anti-BLM antibodies. Consistent with previous reports, BLM protein level was low in G1 phase, rose significantly in S phase and peaked in G2/M phase. In contrast, the level of RMI1 remained fairly constant across different cell cycle phases (Figure 1B).

### 2.2. Accumulation and Phosphorylation of RMI1 at Mitosis

**Figure 2:**
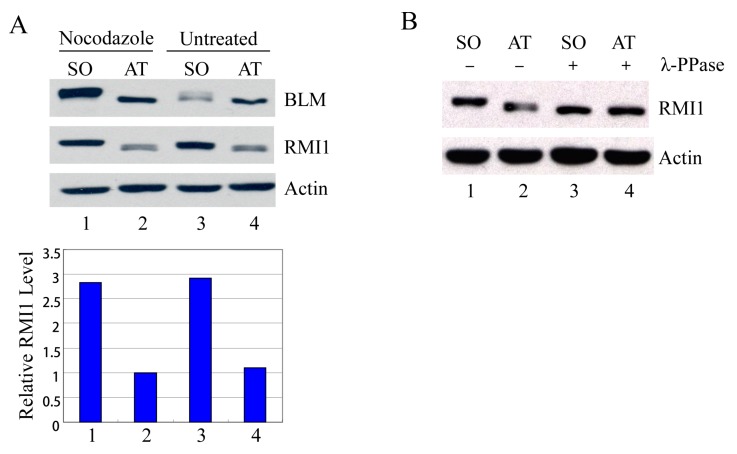
RMI1 accumulates and is phosphorylated in mitosis. (**A**) Mitotic cells were harvested by mechanical shake-off from the nocodazole-arrested HeLa cells and untreated asynchronous HeLa cells. Attached cells were considered as interphase cells. Upper panel: Total cell lysates were analyzed by Western blot; Lower panel: The relative RMI1 protein levels were calculated according to their densitometry readings, which were normalized by the corresponding Actin bands; (**B**) Nocodazole-treated cell lysates were subjected to the λ-phosphatase treatment or not. SO: shake-off cells; AT: attached cells. Numbers under the panels stand for the lane number.

Usually the percentage of mitotic cells is very low in an asynchronous culture due to the brevity of M phase, and the sedimentation velocity of G2 and M cells is similar. Therefore, it is impossible to distinguish M-phase cells from G2-phase cells by centrifugal elutriation. To examine mitotic cells specifically, we treated HeLa cells with nocodazole, an inhibitor of microtubule assembly, to block cells at mitosis, and then manually shook mitotic cells off from nocodazole-arrested cultures. Additionally, to avoid drug effects, we also shook mitotic cells off from asynchronous HeLa cells. These highly enriched populations of mitotic cells were collected and examined by Western blot analysis. Compared to the attached non-mitotic cells, the RMI1 proteins levels are nearly three-fold higher in mitotic cells (Figure 2A). Moreover, like BLM, an almost completely shifted band of RMI1 protein was observed in nocodazole-treated cells. The markedly increased RMI1 protein level and the slow migrating band were also observed in mitotic cells harvested by only mechanical means from the asynchronous HeLa population (Figure 2A, lane 3). These results indicate that post-translational modifications of RMI1 indeed occur during mitosis. Next, we utilized lambda protein phosphatase (λ-PPase) to test whether this band retardation was caused by phosphorylation. The slower migration was eliminated by treatment of the cell lysates with λ-phosphatase (Figure 2B), confirming the phosphorylation of RMI1 in M phase.

### 2.3. Mapping the Mitotic Phosphorylation Sites of RMI1

**Figure 3:**
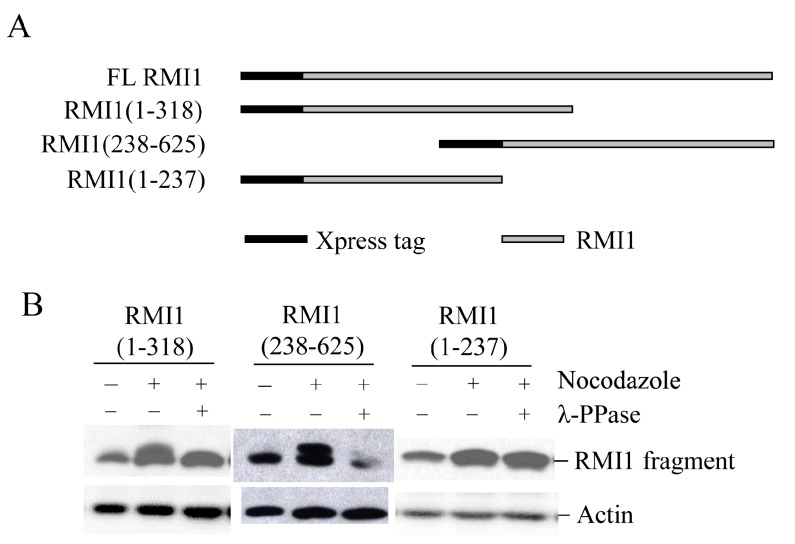
Mapping RMI1 mitotic phosphorylation sites. (**A**) Schematic representation of full-length (FL) Xpress-RMI1 and truncated fragments; (**B**) 293T cells were transfected with the constructs shown above. Thirty-two hours later, cells were treated with nocodazole or not for 16 h prior to harvest. Extracted proteins were treated with λ-phosphatase or not as shown, then resolved by 8% polyacrylamide gel and blotted with anti-Xpress antibody.

To identify the mitotic phosphorylation site(s) on RMI1, initially we generated two overlapped RMI1 fragments as shown in Figure 3A, RMI1 (1–318 amino acid) and RMI1 (238–625 amino acid). Each fragment was fused with amino-terminal Xpress tag and transiently expressed in 293T cells, and the cells were subject to nocodazole treatment. After treating cell lysates with λ-phosphatase or not, the cell lysates were analyzed by Western blotting. As shown in Figure 3B, an up-shifted band was observed on both truncated RMI1 fragments, which was eliminated by λ-phosphatase treatment. These results suggest that the mitotic RMI1 phosphorylation site(s) could be on the overlapping region of RMI1 (238–318) or different regions on each fragment. To narrow down the range, we deleted the overlapping region from the RMI1 (1–318) fragment, resulting in a shorter (1–237) amino-terminal fragment (Figure 3A) and did the same experiment described above. As shown in Figure 3B, no band shift was observed on the RMI1 (1–237) fragment. Taken together, these results suggest the presence of phosphorylation site(s) within the region of residues 238–625 and more likely between the residues of 238 and 318.

### 2.4. RMI1 is Phosphorylated at Serine 284 and 292 during Mitosis

**Figure 4:**
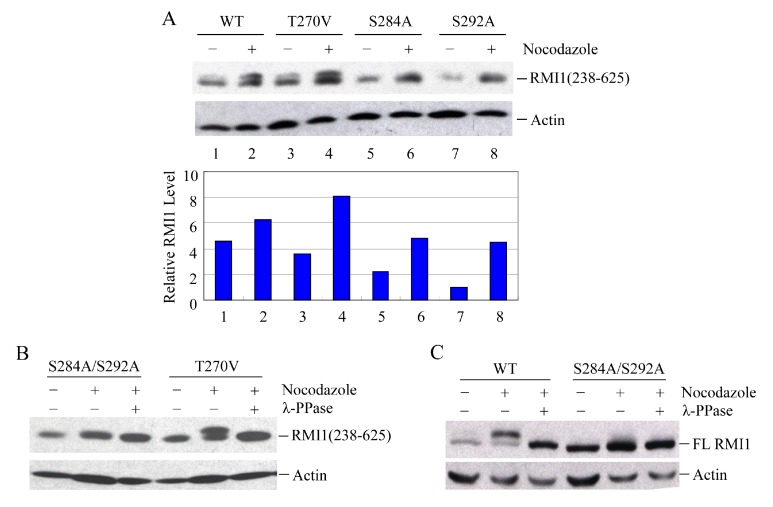
Ser284 and Ser292 are RMI1 mitotic phosphorylation sites. The indicated RMI1 mutants were transiently transfected into 293T cells followed by nocodazole treatment or not for 16 h. Ectopic expression of wild type (WT), T270V, S284A or S292A (**A**); or S284A/S292A, T270V mutation of RMI1 (238–625) fragment (**B**); (**C**) Ectopic expression of full-length wild-type RMI1 or S284A/S292A mutant. Nocodazole treatment and phosphatase treatment were applied as indicated. Quantified relative RMI1 protein levels are shown under the corresponding blots.

To identify the phosphorylated residues on RMI1, we used the Scansite database to analyze RMI1 protein. With medium stringency, there are three candidate residues in the region of 238–318aa: Thr270, Ser284 and Ser292. Since the fragment RMI1 (238–625) showed the clear retarded phosphorylation band, we initially generated the mutation constructs carrying Thr270 to Val (T270V), or Ser284 to Ala (S284A), or Ser292 to Ala (S292A) on the RMI1 (238–625) fragment. The three mutant constructs and wild type RMI1 (238–625) fragment were ectopically expressed in 293T cells, which were then arrested in mitosis by overnight nocodazole treatment. Compared to WT RMI1 and the T270V mutation, the phosphorylation was significantly reduced by the S284A and S292A mutations (Figure 4A). Subsequently, we mutated both Ser284 and Ser292 into alanine (S284A/S292A) on the RMI1 (238–625) fragment and found that phosphorylation of this mutant protein was totally abrogated (Figure 4B), suggesting that Ser284 and Ser292 are the mitotic phosphorylation sites on RMI1. Furthermore, we generated a full-length Xpress-RMI1 containing the double mutations S284A/S292A. Similar results were obtained (Figure 4C), but the shifted band was not completely abolished as the double S-A mutant in the RMI1 (238–625) fragment did, suggesting other minor mitotic phosphorylation site(s) might exist. These results confirmed that Ser284 and Ser292 are the predominant phosphorylation sites of the endogenous RMI1 protein during mitosis.

### 2.5. Phosphorylation of RMI1 at Ser284 and Ser292 Does not Disrupt the Integrity of BTR Complex in Mitosis

**Figure 5:**
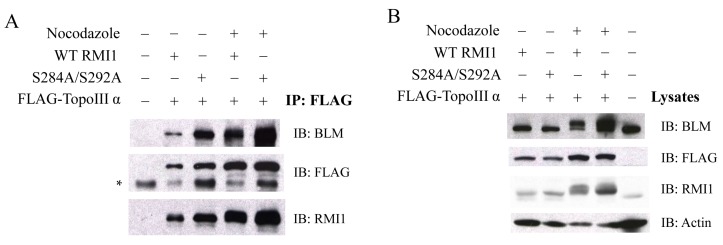
Phosphorylation of RMI1 at Ser284 and Ser292 does not disrupt the interaction between RMI1, TopoIIIα and BLM. 293T cells were transfected with FLAG-tagged Topo III**α** plus Xpress-tagged WT or phospho-mutant (S284A/S292A) RMI1, or GFP expression construct to monitor transfection efficiency, and serve as a negative control (all “−”). Thirty-two hours after transfection, the cells were subjected to nocodazole treatment for 16 h or not as indicated. (**A**) Whole cell extracts were incubated with FLAG-affinity gel. The precipitates were immunoblotted with anti-RMI1, anti-BLM and anti-FLAG antibodies. * Non-specific bands; (**B**) 40 μg of whole cell lysates were analyzed by western blot indicating the input proteins in IP.

Given that both RMI1 and BLM are hyper-phosphorylated in the presence of microtubule destabilization agents, a direct question is whether these post-translational modifications could lead to the dissociation of the BTR complex. To address this question, we co-expressed FLAG-TopoIIIα with the WT or phospho-mutant (S284A/S292A) Xpress-RMI1 in 293T cells. Mitotic cells were accumulated via nocodazole treatment. Then, co-immunoprecipitation (co-IP) was performed to test whether phosphorylated RMI1 (WT) and/or unphosphorylated RMI1 (phospho-mutant) and endogenous BLM protein could be co-immunoprecipitated with Topo IIIα by anti-FLAG beads. As shown in Figure 5, the two types of RMI1 and endogenous BLM were found to associate with TopoIIIα regardless of cell cycle stage. These results demonstrate that the RMI1 phosphorylation status does not interfere with its association with BLM and TopoIIIα, suggesting that phosphorylated RMI1 may still function together with BLM and TopoIIIα during mitosis.

### 2.6. CDK1 Kinase Shares the Responsibility with MPS1 for Mitotic RMI1 Phosphorylation

**Figure 6:**
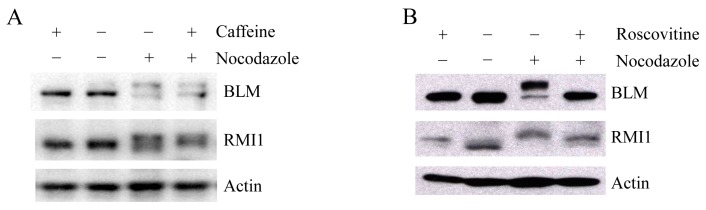
Mitotic RMI1 phosphorylation is partly reversed after roscovitine treatment. HeLa cells were left untreated or treated with nocodazole for 18 h. Thereafter, caffeine (**A**) or roscovitine (**B**) was added for 2 h. Cell lysates were analyzed by Western blotting.

Upon mitosis blocking agents, phosphorylation of BLM at Ser-144 and RMI2 at Ser-112 is completely dependent on the kinase MPS1, while RMI1 phosphorylation only partially depends on MPS1 [22], suggesting another kinase may be also responsible for mitotic RMI1 phosphorylation. In addition to MPS1, three other kinases, ATM, ATR and CDK1, have been reported to phosphorylate BLM proteins [6,24]. To address whether mitotic RMI1 could be a substrate of ATM/ATR or CDK1, we utilized their specific inhibitors, caffeine or roscovitine, to abolish the kinase activity of ATM/ATR or CDK1, respectively. The nocodazole-arrested HeLa cells were treated with caffeine (Figure 6A) or roscovitine (Figure 6B). As previously reported, mitotic BLM phosphorylation was partially reversed by caffeine and completely abolished by roscovitine treatment (Figure 6). In contrast, caffeine had little effect on RMI1 phosphorylation, and partial reversion of RMI1 phosphorylation was observed by roscovitine treatment. These results suggest that CDK1 but not ATM or ATR might contribute to the mitotic phosphorylation of RMI1.

## 3. Discussion

RMI1/BLAP75 is an indispensible component of the BLM-Topo IIIα-RMI1-RMI2 (BTR) complex. It has been well established that RMI1 greatly stimulates the BLM-Topo IIIα-mediated double Holliday junction (dHJ) dissolution in homologous recombination, leading exclusively to non-crossover products to prevent sister chromatid exchanges (SCEs) [25,26]. Interestingly, more and more evidence has shown that BLM and RMI2 are required for the maintenance of chromosome stability at the spindle assembly checkpoint (SAC) [21,22]. Moreover, one interesting observation is that the BLM-Topo IIIα-RMI1 complex localizes to DNA bridges and lagging chromatin during anaphase [27]. These studies implicate RMI1 might also play a role in mitosis.

Firstly, we investigated RMI1 regulation in the cell cycle. Unlike the fluctuating BLM protein levels, there is no apparent alteration for the relative amount of RMI1 protein during the cell cycle progression (Figure 1). However, when we specifically isolated the mitotic cells, we found that the RMI1 protein level increased significantly in the brief M phase. These results suggest RMI1 might have important functions during mitosis. Furthermore, consistent with the recent observation [22], RMI1 is subject to phosphorylation not only upon mitotic interfering agents, but also during undisturbed mitosis (Figure 2), which indicates RMI1 might have mitosis-specific functions.

In eukaryotic cells, the accurate transmission of genetic information relies on cell cycle checkpoints that monitor DNA replication status and accurate chromosome segregation [28]. Among those, the spindle assembly checkpoint (SAC) controls the distribution of chromosomes at mitosis by delaying the onset of anaphase until all chromosomes have properly attached to the mitotic spindle. SAC defects lead to chromosome instabilities such as aneuploidy. Disruption of the mitotic spindle by microtubule poisons, such as nocodazole, activates the SAC and arrests the cells before anaphase onset. Previous studies demonstrated that both phosphorylation of BLM at S114 and phosphorylation of RMI2 at S112 are essential to maintain mitotic arrest upon SAC activation [21,22]. Since RMI1 associates with BLM regardless of their phosphorylation status at mitosis, it is conceivable that mitotic RMI1 phosphorylation may have similar roles as mitotic BLM phosphorylation in spindle checkpoint. To elucidate the biological significance of RMI1 phosphorylation, we firstly tried to identify the RMI1 phosphorylation sites. Fragmentation analysis demonstrated that the phosphorylation sites probably resided within amino acids 238 to 318 of RMI1. Protein motif analysis indicated that three amino acids, Thr270, Ser284, and Ser292, in this region were leading candidates for phosphorylation. By using phosphorylation mutants, we found simultaneous mutation of Ser284 and Ser292 in the RMI1 (238–625) fragment diminished the phosphorylation of RMI1 at mitosis, suggesting these two sites are the major mitotic phosphorylation sites. However, the phospho-mutant RMI1 (S284A/S292A) as full-length form still exhibited some extent of phosphorylation (Figure 4C and Figure 5B), implying the existence of minor phosphorylation sites. Another possibility is that phosphorylation of the residues might require certain domain or the natural folding of the full length RMI1, which was missed in the RMI1 (238–625) fragment. Additionally, these two phosphorylation sites do not appear to be conserved even in metazoans, suggesting that it could be a human/mammal-specific regulation.

Recent work showed that mitotic RMI1 phosphorylation only partially depends on MPS1, which is responsible for both BLM and RMI2 phosphorylation at mitosis [22]. To identify the other possible kinase responsible for RMI1 phosphorylation, we used specific inhibitors to test three potential kinases ATM, ATR, and CDK1, which can phosphorylate BLM. Our results (Figure 6) showed CDK1 is more likely to be that kinase. Moreover, examining two mitotic phosphorylation sites for RMI1, the Ser292 residue in RMI1 is followed by proline, which fits the minimal consensus site requirement (S/T-P) for protein kinase CDK1 and its nearby sequence SPRPK is very close to the full CDK1 consensus site (S/T-P-x-K/R) [29]. However, only partial reversion of RMI1 phosphorylation was observed by roscovitine treatment, which could be ascribed to the effect of roscovitine alone. When comparing to untreated cells, RMI1 protein band also up-shifted in the cells only treated with roscovitine, implying roscovitine alone could induce RMI1 phosphorylation. Therefore, CDK1 kinase could be involved in mitotic RMI1 phosphorylation.

## 4. Materials and Methods

### 4.1. Cell Culture

HeLa and 293T cells were cultured in Dulbecco’s modified Eagle’s medium (DMEM) supplemented with 10% fetal bovine serum (FBS) at 37 °C in a humidified incubator with 5% CO_2_. Transfection of DNA was carried out with Lipofectamine 2000 (Invitrogen, Carlsbad, CA, USA) according to the manufacturer’s instructions.

### 4.2. Chemicals

Nocodazole (Sigma, St. Louis, MO, USA) was resuspended in DMSO to stock concentrations of 1 mg/mL and used at the dilution of 1:2000. Caffeine and roscovitine (Sigma) were resuspended in DMSO to a stock concentration of 100 and 75 mM respectively, and used at the respective dilutions 1:50 and 1:1000.

### 4.3. Protein Phosphatase Treatment

Cells were washed with PBS and subjected to protein extraction. 60 µg of total protein extracts was diluted in phosphatase buffer, MnCl_2_ and water to 25 µL of total volume and incubated in the presence or absence of 600 units of λ-phosphatase (New England Biolabs, Ipswich, MA, USA) for 1 h at 30 °C.

### 4.4. Recombinant DNA

The expression construct, pMal-C2-RMI1, containing the full-length RMI1 was provided by Weidong Wang (NIH/NIA). The wild-type Xpress-RMI1 construct was generated by subcloning the full-length RMI1 from pMal-C2-RMI1 in-frame into the mammalian expression vector pcDNA4/HisMaxC (Invitrogen). To change the selection marker from zeocin to neomycin, the coding sequence of Xpress-BLAP75 was subcloned into another expression vector pcDNA3.1(+). For RMI1 (1–318) deletion mutant, pcDNA3.1-Xpress-RMI1 was digested by StuI and SalI to delete 921 bp of RMI1 and re-ligated the backbone to obtain partially deleted RMI1. Similarly, for RMI1 (1–237) mutant, pcDNA3.1-Xpress-RMI1 was cut by EcoNI and SalI; for RMI1 (238–625) mutant, pcDNA3.1-Xpress-RMI1 was cut by EcoRI and EcoNI. All internal deletion constructs were verified by sequencing.

To construct a FLAG-tagged Topo IIIα expressed in mammalian cells, the full-length Topo IIIα was digested by NaeI and XhoI from pCMV-Sport6-Top3a (OpenBiosystems, Lafayette, CO, USA), and inserted into pCMV-Tag2A vector (Stragagene,La Jolla, CA, USA) that was digested with EcoRV and XhoI.

### 4.5. Site-Directed Mutagenesis

Conversion of Thr270, Ser284 and Ser292 in RMI1 to Val270, Ala284 and Ala292, herein designated RMI1-T270V, RMI1-S284A and RMI1-S292A, was performed using the Stratagene QuickChange site-directed mutagenesis kit. pcDNA3.1-Xpress-RMI1 was used as template and oligonucleotide primers (sense primers) as follows:

RMI1-T270V: 5′-CCTCAGAACGATGTTTCGTCACAGGTAGTTCCTC-3′

RMI1-S284A: 5′-CCCACAAGACAGTCAGCTTTTGAGCCAG-3′

RMI1-S292A: 5′-GAGCCAGAATTTGTTATTGCTCCAAGACC-3′

DNA construct containing single mutant was then used as PCR template to generate RMI1-S284A/S292A double mutant. All generated plasmids were verified by DNA sequencing.

### 4.6. Western Blotting and Antibodies

Cells were collected and lysed with 0.5% Nonidet-P40 buffer containing protease inhibitors on ice for 15 min. Total protein concentration was determined using a BCA protein assay kit (Beyotime Biotechnology, Shanghai, China). Thirty-micrograms total protein of each sample was loaded onto polyacrylamide gels and then transferred to polyvinylidene difluoride (PVDF) membranes (Millipore, Billerica, MA, USA). The primary antibodies used are as follows: rabbit anti-RMI1 antibody (Proteintech, Wuhan, Hubei, China), rabbit anti-BLM (Abcam, Cambridge, UK), mouse anti-Xpress (Invitrogen), mouse anti-Flag M2 antibody (Sigma) and mouse anti-Actin antibody (Chemicon, Temecula, CA, USA).

### 4.7. Immunoprecipitation

293T cells were co-transfected with FLAG-tagged TopoIIIα and Xpress-tagged WT or phospho-mutant (S284A/S292A) RMI1. At the same time, a GFP expression construct was solely transfected into 293T cells to monitor transfection efficiency and serve as a negative control for co-IP. Thirty-two hours after transfection, the cells were subjected to nocodazole treatment for 16 h or not. Then cells were harvested and lysed with 1% Nonidet-P40 buffer with protease inhibitors. The cell lysates containing 1 mg total protein were incubated with 25 µL anti-FLAG M2 beads (Sigma) in immunoprecipitation (IP) buffer (50 mM Tris-HCl, pH 8.0; 150 mM NaCl; 10 mM NaF; 1% Nonidet-P40; 1 mM Na_3_VO_4_; 1 mM DTT and protease inhibitor mixture) at 4 °C for 3 h in a total reaction volume of 1 mL. The beads were washed three times with 1 mL IP buffer. The precipitated proteins were eluted by boiling the beads in 2 × SDS-loading buffer for 10 min. Proteins in the supernatant were separated by 8% polyacrylamide gels and detected by western blotting with the indicated antibodies. Thirty micrograms of cell lysates was used as the input control.

## 5. Conclusions

In summary, we revealed that RMI1 protein level is markedly higher in M phase than in other cell cycle phases, in which it is relatively constant. Moreover, RMI1 is phosphorylated during mitosis and the primary phosphorylation sites are Ser284 and Ser292. In the light of our findings, it is likely that RMI1 may play an important role in mitosis. Further studies are required to elucidate the mitotic function of RMI1.
